# Genetic analysis and chromosome mapping of resistance to *Fusarium oxysporum* f. sp. *niveum* (FON) race 1 and race 2 in watermelon (*Citrullus lanatus* L.)

**DOI:** 10.1007/s11032-015-0375-5

**Published:** 2015-08-29

**Authors:** Yi Ren, Di Jiao, Guoyi Gong, Haiying Zhang, Shaogui Guo, Jie Zhang, Yong Xu

**Affiliations:** National Engineering Research Center for Vegetables, Beijing Academy of Agriculture and Forestry Sciences, Key Laboratory of Biology and Genetic Improvement of Horticultural Crops (North China), Ministry of Agriculture, Beijing, People’s Republic of China; Key Laboratory of Urban Agriculture (North), Ministry of Agriculture, Beijing, People’s Republic of China; Beijing Key Laboratory of Vegetable Germplasm Improvement, Beijing, People’s Republic of China; Institute of Vegetables and Flowers, Chinese Academy of Agricultural Sciences, Beijing, People’s Republic of China; Beijing University of Agriculture, Beijing, People’s Republic of China

**Keywords:** Watermelon, Fusarium wilt, QTL

## Abstract

Fusarium wilt (FW) caused by *Fusarium oxysporum* f. sp*. niveum* (FON) is the major soilborne disease of watermelon (*Citrullus lanatus* L.). The development and deployment of resistant cultivars is generally considered to be an effective approach to control FW. In this study, an F8 population consisting of 103 recombinant inbred lines derived from a cross between the cultivar 97103 and a wild accession PI 296341-FR was used for FON race 1 and race 2 fungal inoculations. One major QTL on chromosome 1 for FON race 1 resistance was detected with a logarithm of odds of 13.2 and explained phenotypic variation *R*^2^ = 48.1 %; two QTLs of FON race 2 resistance on chromosomes 9 and 10 were discovered based on the high-density integrated genetic map we constructed. The nearest molecular marker should be useful for marker-assisted selection of FON race 1 and race 2 resistance. One receptor kinase, one glucan endo-1,3-β-glucosidase precursors and three acidic chitinase located in the FON-1 QTL genomic region. In *Qfon2.1* QTL region, one lipoxygenase gene, five receptor-like kinases and four glutathione S-transferase genes are discovered. One arginine biosynthesis bifunctional protein, two receptor kinase proteins and one lipid-transfer protein located in *Qfon2.2* QTL region. Based on SNP analysis by using 20 re-sequenced accessions of watermelon and 231-plant F_2_ population generated from Black Diamond × Calhoun Grey, we developed a SNP marker Chr1SNP_502124 for FON-1 detection.

**Electronic supplementary material:**

The online version of this article (doi:10.1007/s11032-015-0375-5) contains supplementary material, which is available to authorized users.

## Introduction

China is the largest producer and consumer of watermelon, with an annual production of about 70 million metric tons in 2012 (http://faostat3.fao.org, verified February 24, 2015). Watermelon production in the USA has doubled from 1980 to over 1.77 million metric tons in 2012. Fusarium wilt (FW) of watermelon was the second Fusarium wilt disease reported following the first reported case in cotton (Martyn 2012). In the early 1890s, a serious wilt disease of watermelon in the southern USA caused significant damage. By 1899, similar wilt diseases of muskmelon and cucumbers were reported from OH and CT (Martyn 2012). Many years of domestication and artificial selective breeding for desirable fruit qualities, such as high sugar content and fruit color, resulted in many modern watermelon cultivars with a narrow genetic base and susceptibility to Fusarium wilt (Levi et al. 2001; Lambel et al. 2014). Today, almost none of the Chinese watermelon commercial hybrids have any Fusarium wilt resistance for race 1 and race 2.

The Cucurbitaceae family is affected by several vascular wilts caused by formae speciales of *Fusarium oxysporum* that are generally considered host specific (Martyn 2012). Watermelon FW caused by *F. oxysporum* f. sp*. niveum* (FON) is a major soilborne disease. FW in China has caused significant damage to the watermelon crop production and led to the yield loss of several susceptible commercial cultivars. There are four described pathogenic FON races: 0, 1, 2 and 3 (Martyn and Bruton 1989; Martin and Netzer 1991; Zhou et al. 2010). In China, FON race 1 is the most widespread (Xu et al. 2000), but in the last two decades, FON race 2 has become more prominent around the world. In 1991, an inbred line (PI 296341-FR) that is highly resistant to race 2 was first released (Martyn and Netzer 1991). According to Netzer and Weintall (1980), a dominant gene conditioned the FON race 1 resistance, but it is also affected by modifying gene(s). Resistance to FON race 2 was governed by at least one recessive pair of genes, but a dominant gene from a susceptible parent was epistatic over the recessive gene for resistance in the citron-type PI 296341-FR (Martyn 2012). As a consequence, transferring the high level of FON race 2 resistance from wild citron-type PI 296341-FR into commercial cultivars has proven difficult. Recently, we developed a diploid watermelon hybrid SY630 with the PI 296341-FR resistance. SY630 is a pollinating parent for seedless watermelon production, considering its closer genetic relationship with commercial cultivars than PI 296341-FR, transferring the resistance FON race 1 and race 2 from semi-wild SY630 into commercial cultivars appears to be feasible, but still difficult to break the linkage drag.

FON can survive in soil as saprophyte for many years (Notz et al. 2002). Therefore, watermelons can only be replanted in locations with infected soil, after FON has been eradicated by preplant treatments with soil fumigants. However, the most effective soil fumigant, methyl bromide, has been phased out, and FON can transform itself into thick-walled chlamydospores, highly resistant to chemical fumigation (Lin et al. 2009), leaving hardly any means to control soilborne FON safely, economically and effectively. An alternative is to graft watermelon onto various other cucurbit root stocks such as *Cucurbita* spp. and *Lagenaria* spp. It is effective in controlling Fusarium wilt of watermelon, but it greatly increases production costs, and there is a possibility of altered horticultural characteristics of the cultivars used as scions (Lu et al. 2014). Another alternative is to manage Fusarium wilt disease by breeding resistant lines. Planting resistant cultivars will not only increase the quality and yield of watermelon, but also reduces the use of fungicides.

Compared to traditional phenotype-based selection, marker-assisted selection (MAS) can be an effective method to select quantitative trait loci (QTL) associated with disease resistance and other economically important traits. Although several watermelon germplasms resistant to Fusarium wilt have been released, almost none of the watermelon commercial cultivars have resistance to both FON race 1 and race 2. The lack of molecular markers linked to FON resistance hampers the transfer of the resistance loci to commercial cultivars. In 2013, we developed three molecular markers tightly linked to FON race 1 resistance at one flanking region based on an F_2_ segregating population derived from a cross between the cultivated resistant male parent ‘Calhoun Gray’ and the susceptible female parent ‘Black Diamond’ (Zhang et al. 2013). Lambel et al. (2014) reported a major QTL associated with FON race 1 in 2014 based on genotyping-by-sequencing (GBS) technology producing 266 single-nucleotide polymorphism (SNP) markers. However, they reported seven QTLs associated with FON race 1 resistance on chromosomes 1 (0.00 and 111 cM), 3 (29.2 cM), 4 (13.8 cM), 9 (47.1 cM) and 10 (41.8 and 76.9 cM). This many QTLs and the large interval for the major QTL on chromosomes 1 present a challenge for the breeders to select FON race 1 resistance. Besides the FON race 1 resistance in both our previous work (Zhang et al. 2013) and Lambel’s et al. (2014) report is from cultivars Calhoun Gray and HMw017, respectively, not from the wild accession PI 296341-FR. To the best of our knowledge, there is no report on the FON race 2 resistance, and no QTL for FON race 1 resistance from the accession PI 296341-FR.

In this study, we identified molecular markers linked to FON race 1 and race 2 resistance QTL and performed candidate gene analysis of the QTL regions, based on the high-density integrated genetic map we recently released which contained 698 simple sequence repeat (SSR), 219 insertion–deletion (InDel), 36 structural variation (SV) and 386 SNP markers (Ren et al. 2012; Sandlin et al. 2012; Ren et al. 2014).

## Materials and methods

### Plant materials

An F8 population consisting of 103 recombinant inbred lines (RILs) derived from a cross between the elite Chinese line 97103 and the US PI 296341-FR was used, each line with 15 plants, for fungal inoculations. Cultivars Sugar Baby and Black Diamond were included as highly susceptible controls, whereas cultivars Charleston Grey, Crimson Sweet and Calhoun Grey were used as resistant controls for FON race 1. Calhoun Grey was also used as the susceptible control for FON race 2, whereas SY630 was used as the resistant control for FON race 2.

### Plant growth and fungal inoculation

For surface sterilization, seeds of each watermelon genotype were immersed in 1.5 % sodium hypochlorite for 20 min and then rinsed in distilled water for 4 h. The clean seeds were then incubated at 30 °C for germination. After 36–48 h, the germinated seeds were sowed in sterilized vermiculite and grown in a greenhouse at 25 °C at 80 % relative humidity. FON race 1 and race 2 were originally isolated from a watermelon plot in Beijing. The isolate was confirmed to be race 1 and race 2 by the use of differential watermelon varieties as described by Martyn and Netzer (1991). The conidial suspensions of race 1 and race 2 were prepared in the potato lactose (PL) liquid culture medium and were adjusted to 5.0 × 10^6^ conidia per ml. Watermelon seedlings with both cotyledons open were root-dipped into the conidial suspension for 15 min, and all seedlings were replanted in sterilized soil in a controlled environment of 28 °C, 95 % RH and a 12-h diurnal light cycle. Cotyledons of susceptible plants began to wilt about 10 days after inoculation; final disease rating was taken 4 weeks after inoculation according to the following scale: 1 = healthy; 2 = less healthy compared to resistant control at early stage but survived 2 weeks after inoculation; and 3 = dead. A rating of 1 was considered resistant, 2 was moderately resistant and 3 was considered susceptible.

### Statistical analysis

The analysis of variance (ANOVA) was conducted using PROC GLM in SAS 8.0 (Littell et al. 1998). The broad-sense heritability (*H*^2^) for each trait was calculated on a per plot basis as *H*^2^ = *σ*_G_^2^/(*σ*_G_^2^ + *σ*_GE_^2^/*n* + *σ*_e_^2^/*nr*), where *σ*_G_^2^, *σ*_GE_^2^ and *σ*_e_^2^ were the variance estimates for genotype, genotype by environment interaction and experimental error; *n* and *r* are the number of environments and replications, respectively. The *H*^2^ confidence intervals (CI) were calculated according to Knapp et al. (1989). The Pearson’s phenotypic correlation coefficients among traits across all environments were calculated on a mean basis using the SAS PROC CORR (Littell et al. 1998).

### Detection of FON race 1 and race 2 resistance QTLs

The 97103 × PI 296341-FR 103 RIL population and parents were evaluated in Beijing (39.48°N, 116.28°E) in a randomized complete block design with three replications in the controlled environment of 28 °C, 95 % RH and a 12-h diurnal light cycle. Disease index was calculated for each replication that contains 15 plants of each recombinant inbred line. In our study, QTLs were calculated using the ICIM software (Li et al. 2007) based on an inclusive composite interval mapping and single-marker analysis. Mixed-model-based inclusive composite interval mapping was carried out by using forward–backward stepwise regression with a threshold of *p* = 0.05 to select cofactors, and the window size set at 10 cM (Li et al. 2008; Zhang et al. 2012). The threshold for declaring the presence of a significant QTL was defined by 1000 permutations at a significance level of *p* = 0.05. The confidence interval calculated by the odds ratio reduced by a factor of 10 was averaged for each of the QTL according to Yang et al. (2007). The final genetic model incorporated significant additive and epistatic effects, as well as their interactions with environments.

### PCR amplification for FON-1 validation test

Polymerase chain reactions (PCRs) were performed in 15 μl volumes containing approximately 20 ng template DNA, 1× buffer, 0.5 units Taq polymerase, 20 ng of forward and reverse primers, 2 mM dNTPs. Optimized PCR thermocycling for SNP marker Chr1SNP_502124 incorporated a denaturation step of 5 min at 94 °C, followed by 35 cycles of 20 s at 94 °C, 20 s at 55 °C, 30 s at 72 °C, and ended with a final 4-min extension step at 72 °C. Subsequently, 3 μl of the PCR product was digested by Taq I restriction enzyme and used for electrophoresis in a 6 % polyacrylamide gel.

## Results and discussion

### Phenotypic performance of the 97103 × PI 296341-FR population following FON-1 and FON-2 inoculation

The elite line 97103 had higher disease index than PI 296341-FR for FON-1 and FON-2 (Table 1). Transgressive segregation was observed in the RIL population for FON-2 resistance, while no shift of the average disease index was detected for FON-1 and FON-2 resistance, which were 0.53 and 0.66, respectively. The heritability estimates (*H*^2^) of the FON-1 and FON-2 resistance traits were relatively high (>95 %) (Table 1). The estimate of σ_G_^2^ was highly significant (*p* < 0.001) for all traits. Significant (*p* < 0.001) genotype by environment interactions (GEI) for traits was also observed. GEI variance was primarily due to differences in the magnitudes of the genetic variances among the environments, rather than a lack of genetic correlations between environments. Significant positive correlations (*p* < 0.001) were observed for the phenotypic and genetic correlations among traits across two environments (Table 1).

**Table 1:** Mean value, variance components and broad-sense heritability for FON-1 and FON-2 disease index of the 97103 × PI 296341-FR watermelon RIL population in two environments (Env a and b)

Traits	Env	Parental lines		RILs			Parameter			
		97103	PI 296341-FR	Mean	Min	Max	Genotype (G)	GEI	*H* ^2^ (%)	95 % CI on *H* ^2^
FON-1	a	1	0	0.5	0	1	0.11 ± 0.016**	0.0016 ± 0.0012*	96.69	95.09–97.77
	b	1	0	0.55	0	1				
FON-2	a	1	0.02	0.66	0	1	0.07 ± 0.01**	0.0024 ± 0.0007*	97.96	97.00–98.62
	b	1	0.01	0.67	0	1				

*GEI* genotype by environment interaction, *CI* confidence intervals

*^,^** Significance at *p* < 0.05 and 0.01, respectively

### Segregations between FON-1 and FON-2 resistance traits of RIL population

All 103 RIL, each line with 15 plants, were tested for FON race 1 and race 2 resistance and nearly half of the RILs survived at the 21st day post-inoculation of FON race 1. The expected ratio (1:1) of Resistance:Susceptibility (Table 2) indicates that the resistance for FON-1 is controlled by a QTL with major effect. Only one-quarter of the 103 RIL plants remained healthy with an R:S expected ratio of 1:3 (Table 2), indicating that the resistance for FON-2 might be controlled by two major effect QTLs. Noticeably, 16 out of the 23 that survived at the 21st day post-inoculation of FON race 2 also remained healthy after inoculation of FON race 1. The high ratio (16/23 = 70 %) manifest that if breeders select a FON race 2-resistant plant, it would have a 70 % probability of also getting the FON race 1 resistance trait, but not vice versa, because among the 47 FON race 1-resistant plant, only 34 %(16/47) (calculated from Table 2) of them show resistance to FON race 2. Therefore, a breeding project aiming to select FON-2 resistance would be more effective because of its high positive correlation with FON-1.

**Table 2:** Phenotypic segregation of FON-1 and FON-2 resistance observed in the 97103 × PI 296341-FR watermelon RIL population across two environments

Fungal inoculations	FON race 1	FON race 2	FON race 1 and race 2
Resistant	47	23	16
Susceptible	48	62	85
Medium resistant	8	18	2
Ratio of R:S	47:48	23:62	16:85
Expected ratio of R:S	1:1	1:3	1:7

Plants are classified as: resistant if the disease index is equal or smaller than 0.3; susceptible if the disease index is equal or larger than 0.7; and medium resistant if the disease index is between 0.3 and 0.7

### Detection of QTLs for FON-1 and FON-2 resistance in the 97103 × PI 296341-FR population

**Fig. 1:**
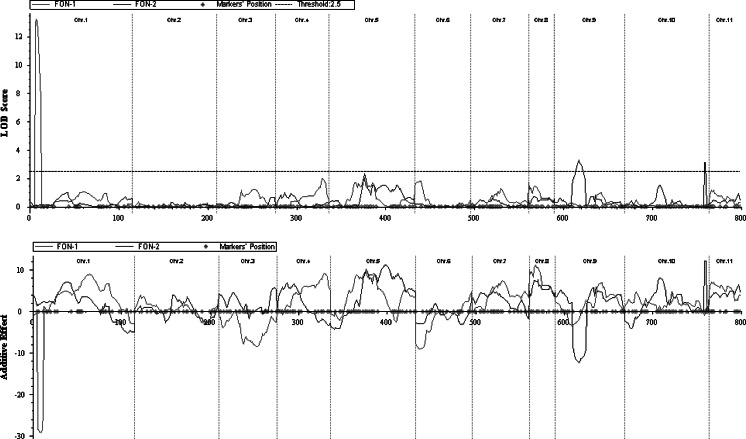
Location, LOD scores and additive effect of QTLs associated with FON race 1 resistance (*red*) on chromosomes 1 and FON race 2 resistance (*green*) on chromosomes 9, 10. (Color figure online)

One major QTL was detected for FON-1 based on inclusive composite interval mapping (Table 3; Fig. 1). The major QTL (*Qfon1.1*) detected on chromosome 1 is located in the interval between 1bin2 (5 cM) and 1bin3 (7.1 cM) for FON-1 trait with maximum LOD = 13.2 and explained 48.1 % of the phenotypic variation. The nearest marker BVWS02309 (655, 755–655, 903 bp on chromosome 1) for FON-1 was detected based on single-marker analysis (Supplementary Table 1). As expected, the additive effect value indicated the wild accession parent PI 296341-FR contributed the favorable alleles at *Qfon1.1* loci (Table 3). This major QTL that we discovered for FON-1 has a much narrower interval (2.1 cM) than the 6.1 cM (2.3–8.4 cM on chromosome 1) reported previously (Lambel et al. 2014) and our previous work (Zhang Yi et al. 2013) which identified with one side flanking markers only. As a consequence, the markers reported in this study will be useful for breeders to select for FON-1 resistance.

**Table 3:** Phenotypic variation (*R* ^2^) explained, additive effect and genetic intervals in bin for each QTL detected

Chr	Trait	Population	QTL name	Left bin	Right bin	Maximum LOD	*R* ^2^ (%)	Additive effect
1	FON-1	RIL	*Qfon1.1*	1Bin1 (0 cM)	1Bin2 (5 cM)	13.2	48.1	−28.47
9	FON-2	RIL	*Qfon2.1*	9Bin8 (19.7 cM)	9Bin9 (28.7 cM)	3.3	13.7	−12.31
10	FON-2	RIL	*Qfon2.2*	10Bin35 (90.2 cM)	10Bin36 (91.6 cM)	3.1	12.5	12.1

Bin positions are based on Ren et al. (2014)

From Lambel’s and our reports, we can draw a conclusion that the FON-1 resistance is from one common site on chromosome 1 in both cultivars (Calhoun Gray and HMw017) and the wild accession watermelon (PI 296341-FR), although their resistance to the FON-1 race varied somewhat. Seedlings of PI 296341-FR were first etiolated but nonetheless survived in the first-week post-fungal inoculations. Calhoun Gray remained healthy during the whole 21 days post-inoculation of FON race 1. It is noticeable that Calhoun Gray showed a higher resistance to FON-1 than did PI 296341-FR, despite the fact that its resistance for FON-1 was originated from African stock citron (WR), and PI 296341-FR was selected from African PI 296341 (Martyn and Netzer 1991).

Two QTLs were detected for FON-2 based on inclusive composite interval mapping (Table 3; Fig. 1). The first QTL for FON-2 (*Qfon2.1*) was identified on chromosome 9 between 9bin8 and 9bin9 with a maximum of LOD = 3.3 and explained 13.7 % of the phenotypic variation. The second QTL for FON-2 (*Qfon2.2*) was identified on chromosome 10 located between 10bin35 and 10bin36 with a maximum LOD = 3.1 and explains 12.5 % of the phenotypic variation. As expected, the allele from the wild accession parent PI 296341-FR contributed to FON-2 resistance at QTL *Qfon2.1*, while the parental cultivar 97103 was associated with FON-2 resistance for the detected QTL *Qfon2.2*. This phenomenon indicates that transgressive inheritance may happen for FON-2 resistance in the RIL population which can explain why some RIL lines have a higher resistance than PI 296341-FR.

### Pathogenesis-related genes in FON-1 QTL

Based on the whole genome sequence of the east Asia watermelon cultivar 97103 we reported (Guo et al. 2012), the interval of the major QTL for resistance to FON race 1 contained 84 genes. According to gene prediction and gene function annotation, the candidate genes for FON race 1 in the mapping region were analyzed, several of which could be useful for future study for resistance to FON race 1 in watermelon. One receptor kinase (Cla004916), one glucan endo-1,3-β-glucosidase precursors (Cla004990) and three acidic chitinase (Cla004914, Cla004920 and Cla004921) located in the FON-1 QTL and flanking 1 M genomic region. Mazzeo et al. (2014) reported that the pathogenesis-related (PR) protein family, glucan endo-1,3-β-glucosidase precursors and acidic chitinase were present in higher amounts in roots of the susceptible tobacco cultivar Monalbo inoculated with *Fusarium oxysporum* f. sp. *radicis*-*lycopersici* (FORL) in comparison with the non-inoculated roots. However, PR proteins are produced during pathogen attack, and their synthesis and accumulation are a common plant defense mechanism against pathogen infection. These enzymes display an inhibitory effect on pathogenic fungal growth by degrading chitin and β-glucan that are components of fungal cell walls (Van Loon et al. 2006). Therefore, future research works are required to identify whether these PR genes are associated with resistance to FON-1 in watermelon.

### Resistance- and susceptibility-related genes to Fusarium wilt disease in FON-2 QTL

In the *Qfon2.1* QTL region, one lipoxygenase (Cla014845) gene, five receptor-like kinases (Cla014853, Cla014955, Cla014972, Cla014725 and Cla014728) and four glutathione S-transferase (Cla014674–Cla014677) genes are discovered. Lipoxygenase proteins are known to be involved in biotic and abiotic defense mechanisms of plants (Yan et al. 2013), while the receptor kinases play important roles in pathogen recognition and in growth and defense of plants. Glutathione S-transferases are involved in the glutathione-dependent detoxification pathway that plays a crucial role in detoxification of xenobiotics. In tomato roots inoculated with FORL, the infected resistant (Momor) roots showed a higher amount of glutathione S-transferase compared to the susceptible (Monalbo) genotype (Mazzeo et al. 2014). From this result, it could be inferred that the four glutathione S-transferase might participate in conferring resistance to fungal infection.

In *Qfon2.2*, one arginine biosynthesis bifunctional protein (Cla017879), two receptor kinase proteins (Cla017918 and Cla017919) and one lipid-transfer protein (Cla018045) are present. Up-regulation of arginase activity and its gene expression has been reported in infected resistant roots in tomato (Mazzeo et al. 2014). Transgenic tomato plants in which lipid-transfer protein (LTP) gene is silenced exhibited reduced disease susceptibility to Fusarium wilt (Krasikov et al. 2011). Interestingly, the reduced symptoms observed did not correlate with an altered expression profile for known reporter genes of plant defense (PR-1 and WIPI). This work demonstrates that lipid-transfer protein is required for full susceptibility of tomato to Fusarium wilt (Krasikov et al. 2011). Considering the parental cultivar 97103 was associated with FON-2 resistance for the detected QTL *Qfon2.2*, it is possible that the low expression of the susceptibility-related lipid-transfer protein (Cla018045) contributed to the FON-2 resistance in RIL population.

### Validation test for FON resistance in germplasms and F_2_ population

We developed SNP marker Chr1SNP_502124 (flanking sequences of Chr1SNP_502124 were available at www.icugi.org) for FON-1 detection, based on SNP analysis in the QTL regions. Validity of the SNP marker was tested using 20 re-sequenced accessions of watermelon (Guo et al. 2012) that include all the three subspecies: the cultivar *C. lanatus* spp. *lanatus* L., which represents a group of cultivars, the semi-wild *C. lanatus* spp. *mucosospermus* L. and the wild ‘tsamma’ or ‘citron’ watermelon that naturally thrives in southern Africa. All the 20 re-sequenced accessions match the FON-1 phenotype at FON-1_Chr1SNP_502124 loci (Table 4), suggesting that the accuracy of this marker for use in breeding programs would be high. Although the 20 re-sequenced accessions represent all three types of watermelon, the re-sequenced population size is not large; therefore, we tested the SNP marker in a 231-plant F_2_ population generated from Black Diamond × Calhoun Grey. The phenotype of 229 plants in the F_2_ population at the 21st day post-inoculation of FON race 1 agrees with the prediction based on the genotype of the SNP marker Chr1SNP_502124, indicating that this SNP marker can be used for FON-1 selection effectively. In order to introduce the FON-1 resistance trait into commercial hybrid cultivars, a marker-assisted selection breeding program was carried out using FON-1_Chr1SNP_502124 for selection of FON-1 resistance. All the plants containing SY630 genotype (heterozygote) at the FON-1_Chr1SNP_502124 locus show FON-1 resistance in a 61-plant BC1 population generated from a cross of an elite Chinese line KYF × SY630 (Supplementary Fig. 1). We attempted to identify SNPs in the FON-2 QTL region for race 2 by the same method as we used for the identification of FON-1_Chr1SNP_502124 locus. Because there are only two accessions with FON-2 resistance, we found too many SNPs that are associated with FON-2 resistance. A large population generated from Black Diamond × SY630 is being developed for the identification of SNPs for FON-2 in the *Qfon2.1* and *Qfon2.2* QTL region.

**Table 4:** Accession names and their phenotypes applied for SNP analysis

Accession names	Subspecies	FON-1_Chr1SNP_502124	FON-1 phenotype
97103	Cultivar *C. lanatus* spp. *lanatus* L.	T	S
RZ900	Cultivar *C. lanatus* spp. *lanatus* L.	T	S
RZ901	Cultivar *C. lanatus* spp. *lanatus* L.	T	S
JLM	Cultivar *C. lanatus* spp. *lanatus* L.	T	S
JXF	Cultivar *C. lanatus* spp. *lanatus* L.	T	S
XHBFGM	Cultivar *C. lanatus* spp. *lanatus* L.	T	S
Black Diamond	Cultivar *C. lanatus* spp. *lanatus* L.	T	S
PI 248178	Cultivar *C. lanatus* spp. *mucosospermus* L.	T	S
PI 189317	Cultivar *C. lanatus* spp. *mucosospermus* L.	T	S
PI 482271	Cultivar *C. lanatus* spp. *mucosospermus* L.	T	S
PI 595203	Cultivar *C. lanatus* spp. *mucosospermus* L.	T	S
PI 249010	Cultivar *C. lanatus* spp. *mucosospermus* L.	T	S
PI 482276	Wild *C. lanatus* spp. *citron* L.	T	S
PI 482303	Wild *C. lanatus* spp. *citron* L.	T	S
PI 482326	Wild *C. lanatus* spp. *citron* L.	T	S
PI 500301	Cultivar *C. lanatus* spp. *mucosospermus* L.	T	S
JX2	Cultivar *C. lanatus* spp. *lanatus* L.	G	R
Calhoun Gray	Cultivar *C. lanatus* spp. *lanatus* L.	G	R
Sy904304	Cultivar *C. lanatus* spp. *lanatus* L.	G	R
Sugarlee	Cultivar *C. lanatus* spp. *lanatus* L.	G	R

All the test susceptible (S) accessions’ genotypes are T at 502124 loci, and the resistant (R) genotypes are G

## Electronic supplementary material

Supplementary Fig. 1 Assessment of the molecular marker FON-1_Chr1SNP_502124 for FON-1 resistance in the BC1 population. Lanes 1 and 2 represent susceptible commercial cultivar KYF and resistant parent SY630, respectively. Lanes 3 to 63 represent the 61 genotypes in the BC1 population generated from KYF × SY630. All heterozygous genotypes show FON-1 resistance. (TIFF 664 kb)

Supplementary material 2 (DOCX 18 kb)
