# Supplementary figures and images for: Genetic analysis and chromosome mapping of resistance to Fusarium oxysporum f. sp. niveum (FON) race 1 and race 2 in watermelon (Citrullus lanatus L.)
Source: Mol Breed. 2015 Aug 29;35(9):183. doi: 10.1007/s11032-015-0375-5 (PMC4552779; doi:10.1007/s11032-015-0375-5)

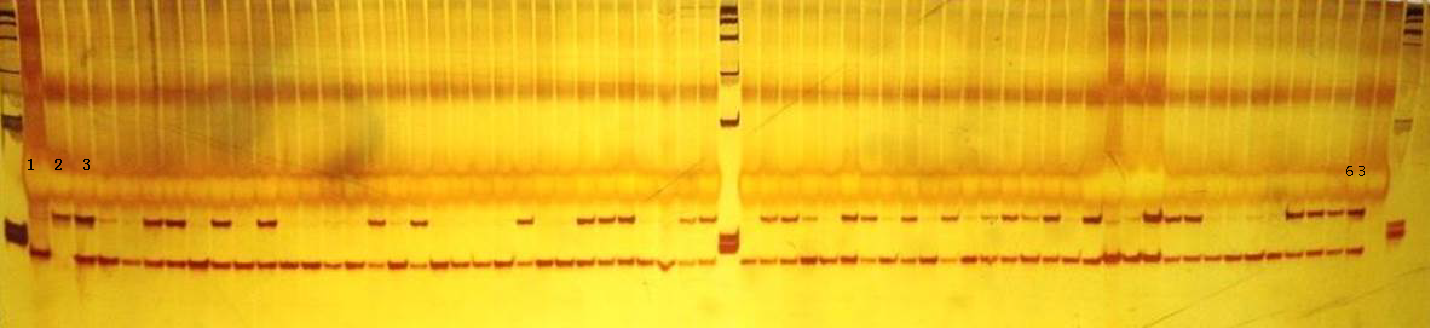

Supplement: Supplementary file 1 — Assessment of the molecular marker FON-1_Chr1SNP_502124 for FON-1 resistance in the BC1 population. Lanes 1 and 2 represent susceptible commercial cultivar KYF and resistant parent SY630, respectively. Lanes 3 to 63 represent the 61 genotypes in the BC1 population generated from KYF × SY630. All heterozygous genotypes show FON-1 resistance. (TIFF 664 kb) [file 11032_2015_375_MOESM1_ESM.tif]
