# Supplementary material for: Genetic analysis and chromosome mapping of resistance to Fusarium oxysporum f. sp. niveum (FON) race 1 and race 2 in watermelon (Citrullus lanatus L.)
Source: Mol Breed. 2015 Aug 29;35(9):183. doi: 10.1007/s11032-015-0375-5 (PMC4552779; doi:10.1007/s11032-015-0375-5)
Supplement: Supplementary file 2 — Supplementary material 2 (DOCX 18 kb) [file 11032_2015_375_MOESM2_ESM.docx]

Supplementary Table1 Names, sequences and physical positions of mapped nearest markers to the QTL peak and developed SNP marker Chr1SNP_502124 for FON-1 detection.

| QTL name | Nearest marker | Marker type | Repeat  motif | Production size | Forward  primer | Reverse  primer | Primer in 97103 genome ^a^ |
| --- | --- | --- | --- | --- | --- | --- | --- |
| *Qfon1.1* | BVWS02309 | SSR | (AG)13 | 149 | AATCTCCACTACAATCCACCAG | TTCCTCCAAACTCATCATTACC | 655755  -655903 |
| *Qfon2.1* | BVWS01133 | SSR | (AAT)6 | 260 | CATCCACCTCAAACTTTAGAAACA | TTCTATTCCCGTCATTTCATTG | 9009787-9010046 |
| *Qfon2.2* | BVWS02333 | SSR | (AAG)5 | 103 | GGGGGTTTTGGTTTCTTGAT | ATGATGTCACCATTACGGGG | 27300996-27301098 |
| *-* | Chr1SNP_502124 | SNP ^b^ | - | 116 | AACACCACCCACTTTGGAGCTTCG | TTTTAGGGTGAAAATGGGTATTGTA | 502110-502215 |

^a^ The genome physical positions were according to Guo et al (Guo et al. 2012).

^b^ The SNP marker was transformed to dCAPS with Taq I restriction site followed by a normal procedures for PCR and electrophoresis in a 6% polyacrylamide gel in validation test.
